# Liquid Chalk Is an Antiseptic against SARS-CoV-2 and Influenza A Respiratory Viruses

**DOI:** 10.1128/mSphere.00313-21

**Published:** 2021-06-16

**Authors:** Julie L. McAuley, Joshua M. Deerain, William Hammersla, Turgut E. Aktepe, Damian F. J. Purcell, Jason M. Mackenzie

**Affiliations:** a Department of Microbiology and Immunology, University of Melbourne, at the Peter Doherty Institute for Infection and Immunity, Parkville, VIC, Australia; b Urban Climb, Collingwood, VIC, Australia; University of Maryland School of Medicine

**Keywords:** SARS-CoV-2, COVID-19, influenza A virus, norovirus, antiseptic, alcohol, noroviruses

## Abstract

The COVID-19 pandemic has impacted and enforced significant restrictions within our societies, including the attendance of public and professional athletes in gyms. Liquid chalk is a commonly used accessory in gyms and is comprised of magnesium carbonate and alcohol that quickly evaporates on the hands to leave a layer of dry chalk. We investigated whether liquid chalk is an antiseptic against highly pathogenic human viruses, including SARS-CoV-2, influenza virus, and noroviruses. Chalk was applied before or after virus, inoculum and recovery of infectious virus was determined to mimic the use in the gym. We observed that addition of chalk before or after virus contact led to a significant reduction in recovery of infectious SARS-CoV-2 and influenza virus but had little impact on norovirus. These observations suggest that the use and application of liquid chalk can be an effective and suitable antiseptic for major sporting events, such as the Olympic Games.

**IMPORTANCE** To restrict the potential transmission and infectivity of SARS-CoV-2, the use of liquid chalk has been a requirement in an active gym setting. However, its effectiveness has not been scientifically proven. Here, we show that the application of liquid chalk before or after virus inoculum significantly impacts recovery of infectious SARS-CoV-2 and influenza viruses but not noroviruses. Thus, our study has shown that the implementation and application of liquid chalk in communal social gym settings is effective in reducing the infectivity of respiratory viruses, and this supports the use of liquid chalk in major sporting events to restrict the impact of COVID-19 on our communities.

## INTRODUCTION

The use and application of hand sanitizers in preventing the spread of infectious microbial diseases are primarily based on the 60 to 80% (vol/vol) proportion of alcohol included within these products (1, 2). The use of hand sanitizers has been an important control measure in limiting the spread of virus during the recent COVID-19 pandemic in some settings (3, 4). Liquid chalk is a product that comprises magnesium carbonate (chalk), 40 to 80% alcohol (generally ethanol, methanol, or isopropanol), water, and sometimes other additives including resins or proprietary materials, depending on the manufacturer. When applied to the hands, the liquid chalk is distributed across the surface of the hand and then dries into a thin chalk layer as the alcohol evaporates. Due to the high percentage of alcohol, liquid chalk has been suggested to act as a hand sanitizer against SARS coronavirus, although this has yet to be proven experimentally. In this study, we investigated and evaluated the application of various liquid chalk products as antiseptics against the spread and transmission of SARS-CoV-2 (the causative agent of the COVID-19 pandemic), influenza A virus (H1N1) (IAV), and norovirus, using the surrogate model of mouse norovirus (MNV).

## RESULTS

### Experimental rationale.

In our experimental design, we investigated two different approaches to represent the scenarios encountered within the gym environment. First, we tested the application of liquid chalk on a surface that already contained the virus, and second, we tested the ability of liquid chalk to prevent transmission once applied to a surface with subsequent addition of the virus. For the virus-first experiments, a volume containing a known titer of virus (SARS-CoV-2, IAV, or MNV) was applied to a plastic surface. Subsequently, a known volume of four commercially available and widely used liquid chalk products was added, smeared to a thin layer, and then allowed to dry. The entire layer was then resuspended in tissue culture medium, and the chalk particulate was removed by centrifugation. The resultant supernatant was then diluted and added to Vero (SARS-CoV-2), MDCK (IAV), or RAW 264.7 cells (MNV), and virus infectivity was measured by performing a 50% tissue culture infectious dose (TCID_50_) assay. For the chalk-first experiments, the exact same procedure described above was performed except that the volume of chalk was applied first and allowed to dry and then the volume of virus was added to the dried chalk.

### Liquid chalk prevents the recovery of infectious SARS-CoV-2.

**FIG 1:**
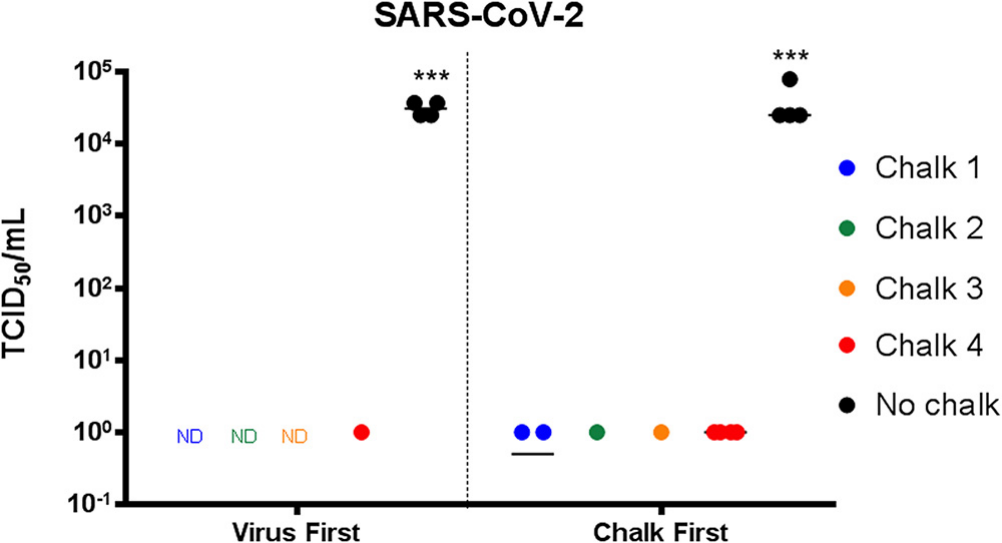
SARS-CoV-2 is rendered noninfectious by gym liquid chalk. All chalks tested significantly reduced the amount of infectious SARS-CoV-2 in the sample compared to the no-chalk control (ND, not detectable) when added either before or after the viral inoculum. (***, *P* < 0.001 compared to no-chalk, one-way analysis of variance [ANOVA]).

As can be observed in Fig. 1, all four liquid chalk products significantly impacted the recovery of SARS-CoV-2 virus from the surface to which it was applied. Intriguingly, chalks 1 to 3 all displayed complete loss of the recovery of virus (to the limit of detection) whereas chalk 4 also had a significant impact but some residual virus could be recovered. Of interest was our observation that the application of liquid chalk before or after virus inoculum had an equal effect on the recovery of SARS-CoV-2 virus. In this assay, the alcohol contained in the liquid chalk product was allowed to evaporate prior to contact with virus, suggesting the dried chalk provided a virucidal activity. To confirm the antiviral effects of alcohols, we treated SARS-CoV-2 virus preparations with differing amounts and types of alcohols (including those commonly found in liquid chalk products). As summarized in Table 1, we observed that all alcohols had a virucidal effect on SARS-CoV-2. Thus, our results indicate that the application and implementation of liquid chalk can be a suitable antiseptic against the transmission of SARS-CoV-2. Liquid chalk itself was not cytotoxic to any of the cell types used in this study at the concentrations shown to be effective in reducing virus recovery (see Fig. S1 in the supplemental material).

**TABLE 1:** Recovery of virus after 15-min exposure to alcohol

Alcohol, % (vol/vol)	Virus (log_10_/ml)			
	SARS-CoV-2		IAV	
	Ethanol	Isopropanol	Ethanol	Isopropanol
80	ND^*a*^	ND	ND	ND
60	ND	ND	5.13	ND
40	ND	ND	6.9	6.4
20	3.4	4.24	7.26	7.23
0		4.57		7.24

a ND, not detectable.

10.1128/mSphere.00313-21.2 FIG S1 Cytotoxic effects of differing dilutions of the liquid chalks used in this study. Vero, MDCK, and RAW 264.7 cells were exposed to differing dilutions of the liquid chalks to determine the cytotoxic effect on the different cell types. As can be observed, only when the liquid chalks were used at a neat (or 100%) dilution did the cells show any adverse or cytotoxic effects. All these dilutions are within the limits used during the treatment studies and indicate that the chalk itself is not contributing to the effects observed. Download FIG S1, TIF file, 0.5 MB. © Crown copyright 2021. 2021 Crown https://creativecommons.org/licenses/by/4.0/ This content is distributed under the terms of the Creative Commons Attribution 4.0 International license.

### Liquid chalk prevents the recovery of infectious IAV.

**FIG 2:**
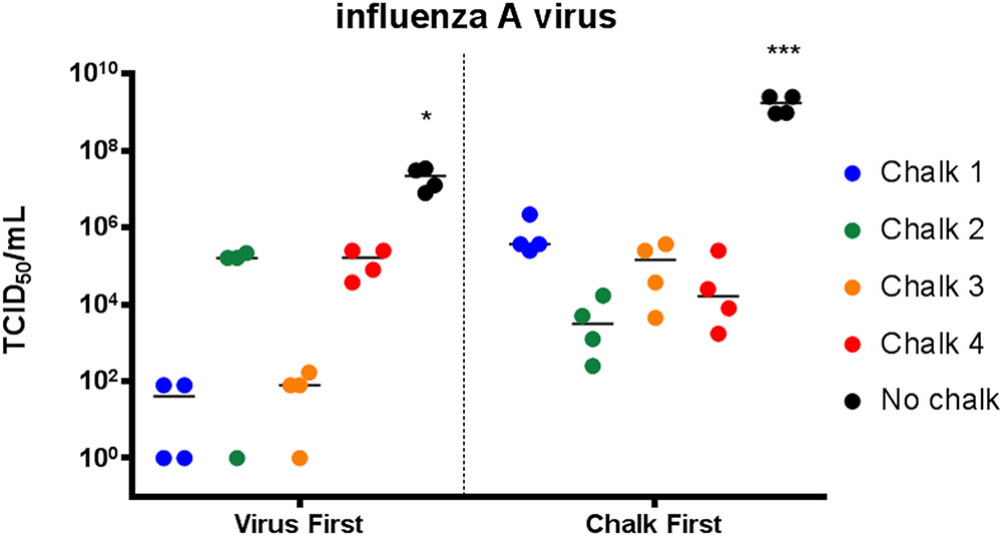
Influenza A virus is significantly inactivated by liquid chalk. When influenza A virus was applied to the surface first, chalk 1 and chalk 3 reduced the amount of infectious virus to nearly undetectable levels (*P* < 0.001 compared to no-chalk control). When chalk was applied first and dried, the recovery of infectious influenza A virus was significantly reduced compared to the chalk control, but markedly more infectious virus remained in the samples treated with chalk 1 and chalk 3 compared to the virus-first samples that underwent the same treatment (data not significant). (*, *P* < 0.05; ***, *P* < 0.001 compared to no chalk, one-way ANOVA).

To further our study, we also tested the antiviral effect of liquid chalk against another highly infectious and pathogenic respiratory viral pathogen, influenza A virus (IAV). The experiments were performed exactly as described above, and the virus TCID_50_/milliliter was titrated on MDCK cells. As can be observed in Fig. 2, all four liquid chalk products were effective in restricting the recovery of IAV compared to SARS-CoV-2. However, for IAV the effect was greater when the chalk was applied to the virus inoculum rather than first chalk and then virus, linking the presence of alcohol as a significant antiseptic component against IAV. As can be observed, application of chalks 1 and 3 reduced the virus recovery by approximately 6 logs (TCID_50_/milliliter) whereas chalks 2 and 4 reduced the recovery by ∼2.5 logs. When chalk was first applied to the surface, all chalks induced an approximately 2- to 4-log decrease in virus recovery. Overall, these results indicate that the application of liquid both to IAV or onto a surface can reduce the recovery of IAV.

### Liquid chalk does not prevent the recovery of infectious norovirus.

**FIG 3:**
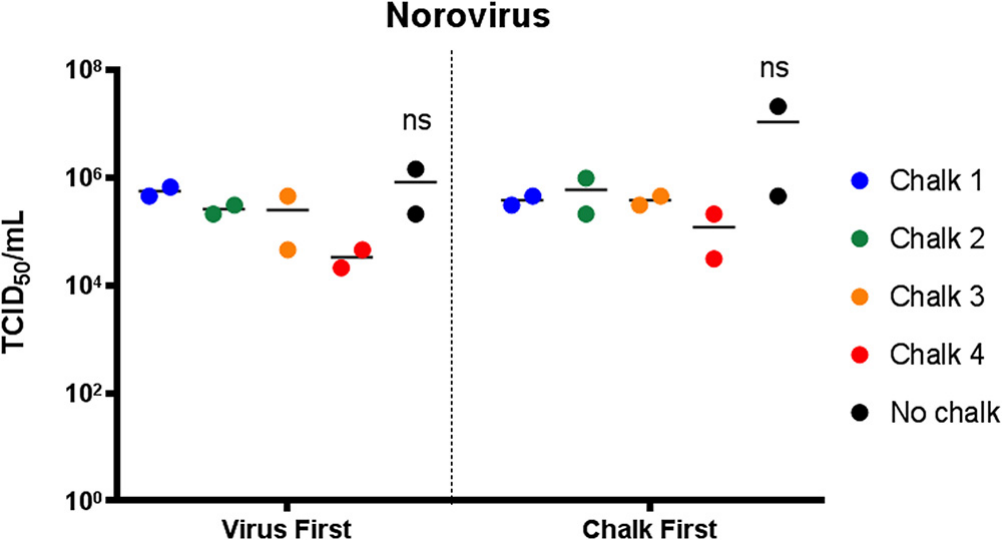
Norovirus remains infectious when exposed to liquid chalk. Norovirus was not rendered noninfectious when treated with gym chalk, regardless of whether the virus was added to dry chalk or chalk was added to virus inoculum (ns, not significant; *P* > 0.05 to no chalk, one-way ANOVA).

As a comparator, we also investigated the ability of liquid chalk to inactivate another highly infectious viral pathogen, norovirus. As human norovirus is difficult to cultivate under laboratory conditions, we utilized the widely appreciated surrogate murine norovirus (MNV) for our studies (5). Again, the experiments were identical to those described above except the viral TCID_50_/milliliter was performed on RAW 264.7 (murine macrophage) cells. Intriguingly, Fig. 3 shows that MNV is relatively resistant to the virucidal properties of the liquid chalk products. We observed that chalks 1 to 3 had very little impact on virus recovery, with a 0.5-log reduction the best that we observed. However, in contrast to SARS-CoV-2 we observed an approximately 1-log reduction upon application of chalk 4. This is interesting as the major difference between MNV and SARS-CoV-2 and IAV is that MNV is a nonenveloped virus, whereas the other two contain a host-derived lipid membrane as their outermost layer. Thus, it would be interesting to identify the constituents of chalk 4 as it was the effective agent against MNV but was observed to be slightly less effective against SARS-CoV-2.

### SARS-CoV-2 and IAV are sensitive to treatment with various concentrations of alcohols.

As different alcohols are the major constituents of liquid chalk, we additionally evaluated the impact of alcohol alone on the recovery of infectious virus. As can be observed in Table 1, exposure of SARS-CoV-2 and IAV to both ethanol and isopropanol is detrimental to the infectiousness of these viruses up to a dilution of 40% (vol/vol) for IAV and 20% (vol/vol) for SARS-CoV2. Given proprietary information regarding the alcohol content of chalks, we can only conjecture that if lower percentages of alcohol than these are present in the liquid chalk, then it is likely that the antiviral activity we observe can be attributed to the chalk component.

## DISCUSSION

There have recently been two press releases about studies on liquid chalk, one using seasonal CoV and not SARS-CoV-2 (6) and the other using SARS-CoV-2 but only a single chalk product (7). Neither study as described in its press release investigated the efficiency against other highly infectious viral pathogens. This is the first report to directly compare two highly infectious respiratory pathogen (SARS-CoV-2 and influenza virus) and fomite-transmitted (norovirus) viruses for their resistance or sensitivity to liquid chalk. Overall, this report is novel and the comparison has not been performed previously. We have observed that the other highly pathogenic respiratory virus influenza A virus is also sensitive to liquid chalk compared with SARS-CoV-2. One of the additional findings that we have observed is that the chalk, once dried, i.e., after the alcohol has evaporated, is still effective against both IAV and SARS-CoV-2. Of interest was that not all chalks were observed to be equally effective yet all contained various amounts of alcohol. Intriguingly, noroviruses, which are sensitive to alcohols (8, 9), are not sensitive to liquid chalk. These observations suggest that it is more than just the alcohol that contributes to resistance or sensitivity under the conditions tested.

To our knowledge, this study is the first to demonstrate antiviral activity of a range of commercially available and commonly used liquid chalks. Given the uncertainty of reopening gyms due to contact transmission from potentially contaminated equipment, our findings that liquid chalks have antiviral activity against SARS-CoV-2 may aid in decision making for reopening gyms in the future. This is important due to the impact of gym closures (due to COVID-19) on personal fitness, professional sports, and particularly mental health and well-being.

## MATERIALS AND METHODS

### Liquid chalks.

Four liquid chalks were randomly selected and obtained from four different commercial sources. Brand identity can be obtained via personal communication from the authors. Publicly available information regarding the composition of each chalk mixture is given in Table 2.

**TABLE 2:** Liquid chalk ingredients

Chalk	Publicly listed ingredients
1	Alcohol, magnesium carbonate, perfume
2	70% alcohol, magnesium carbonate
3	Isopropyl alcohol, magnesium carbonate hydroxide, colophonium, hydroxypropyl cellulose, Styrax benzoin resin
4	Ethanol, magnesium carbonate, water

### Cell culture maintenance and virus stocks.

Vero cells (American Type Culture Collection [ATCC]) were maintained in minimal essential medium (MEM) supplemented with 10% heat-inactivated fetal bovine serum (FBS), 10 μM HEPES, 2 mM glutamine, and antibiotics (100 units/ml penicillin G, 100 μg/ml streptomycin). Madin-Darby canine kidney (MDCK) cells were grown in Roswell Park Memorial Institute (RPMI) medium supplemented with 10% FBS, 2 mM glutamine, and antibiotics. RAW 264.7 cells were maintained in Dulbecco’s modified Eagle’s medium (DMEM) with 10% FBS and 1% GlutaMAX. Cell cultures were maintained at 37°C in a 5% CO_2_ incubator. SARS-CoV-2 isolate hCoV-19/Australia/VIC01/2020 (10) stocks were produced as previously described (11). The influenza A virus isolate, A/Puerto Rico/8/34, was produced as previously described (12). Details of MNV strain CW1 have been described previously (13, 14).

### Cytotoxicity assay 96 nonradioactive cytotoxicity assay (Promega).

Vero, MDCK, and RAW cells were plated in a 96-well plate to 80% confluence. Fifty microliters of each liquid chalk sample was aseptically air dried, resuspended in 500 μl of cell culture medium, and centrifuged at 400 × *g* for 3 min to remove excess chalk particles (this sample is depicted as “neat”). Neat supernatant was 10-fold serially diluted in respective tissue culture medium, added to the 96-well plate containing cells, and incubated at 37°C for 24 h. Following the incubation period, 10 μl of 10× lysis solution was added for 45 min to the control wells to generate a maximum lactate dehydrogenase (LDH) release control. Fifty microliters of supernatant from each sample (in duplicates) was transferred to a fresh 96-well flat- and clear-bottom plate and incubated for 30 min with 50 μl of CytoTox 96 reagent. To end the reaction, 50 μl of stop solution was added to each well and absorbance was recorded at 490 nm (optical density at 490 nm [OD_490_]). Percent cytotoxicity was calculated as 100× [experimental LDH release (OD_490_)/maximum LDH release (OD_490_)].

### Chalk exposure assays.

All SARS-CoV-2 infection cultures were conducted within the high-containment facilities in a Physical Containment Level 3 (PC3) laboratory at the Doherty Institute. For the chalk-first assay, a sample of the liquid chalk was aseptically smeared onto 4 discrete areas covering approximately a 2-cm round surface (approximately 50 μl) on a sterile tissue culture dish and allowed to dry. Fifty microliters of virus inoculum was then applied. Where the inoculum did not absorb into the dry chalk, a slurry of chalk-virus mixture was created using a sterile tip and mixing. Fifteen minutes later, 500 μl infection medium (identical to culture medium but without the presence of sera) was added, and the sample was mixed and collected. Chalk was pelleted at 400 × *g* for 3 min, and then a 50% tissue culture infectious dose (TCID_50_) assay performed on the supernatant as previously described (11, 12). For the virus-first assay, 50 μl of virus inoculum was applied as a drop to 4 discrete areas of a sterile tissue culture dish followed by addition of 50 μl liquid chalk. A sterile tip was used to mix the chalk with virus, and the mixture was left for 15 min. In the same way as the chalk-first procedure, 500 μl infection medium was added and sample was mixed, and then remaining virus present in the sample was quantitated by TCID_50_ assay. For both tests, the no-chalk control received 50 μl infection medium only instead of the liquid chalk.

### TCID_50_ assay.

Known volumes of SARS-CoV-2, IAV, and MNV samples were serially diluted and then added in quadruplicate repeats to washed confluent monolayers of Vero and MDCK cells, for SARS-CoV-2 and IAV, respectively, or 80% RAW cells for MNV in serum-free medium (11, 12). Virus-induced cytopathic effect (CPE) was then microscopically observed 3 days later. The dilution of each sample required to induce CPE in 50% of the wells was then determined by the method of Reed and Muench (15) and recorded as TCID_50_/milliliter.

### Statistical analyses.

Data are representative of 3 biological replicates that included 2 technical replicates and were analyzed by one-way analysis of variance (ANOVA) using GraphPad Prism v8.0 as described in the figure legends.

### Patient and public involvement statement.

We state that patients or the public were not involved in the design, or conduct, or reporting, or dissemination plans of our research.

### Ethical approval information.

No animals or humans were utilized during this study. The infection with SARS-CoV-2 is covered by our Institutional Biosafety Committee approval number 2020/028.

10.1128/mSphere.00313-21.1 TEXT S1 Description of the materials and methods and results contributing to Fig. S1 and S2. Here, we describe the approaches used to determine the cytotoxic effects of different liquid chalk dilutions on the cells used in this study and the effect that different alcohols have on the recovery of infectious SARS-CoV-2 and IAV. We report our results within this supplemental material. Download Text S1, DOCX file, 0.03 MB. © Crown copyright 2021. 2021 Crown https://creativecommons.org/licenses/by/4.0/ This content is distributed under the terms of the Creative Commons Attribution 4.0 International license.

10.1128/mSphere.00313-21.3 FIG S2 Impact of alcohol treatment alone on the recovery of SARS-CoV-2 and IAV. We determined the impact of alcohol treatment alone on each of these viruses as they had shown sensitivity to the alcohol-based liquid chalks. As can be observed, 80% and 60% ethanol affected the infectivity of IAV but 40% did not. Comparatively, isopropanol even at 40% caused a highly significant loss of infectivity. Download FIG S2, TIF file, 0.2 MB. © Crown copyright 2021. 2021 Crown https://creativecommons.org/licenses/by/4.0/ This content is distributed under the terms of the Creative Commons Attribution 4.0 International license.
