# Supplementary figures and images for: Liquid Chalk Is an Antiseptic against SARS-CoV-2 and Influenza A Respiratory Viruses
Source: mSphere. 2021 Jun 16;6(3):e00313-21. doi: 10.1128/mSphere.00313-21 (PMC8265647; doi:10.1128/mSphere.00313-21)

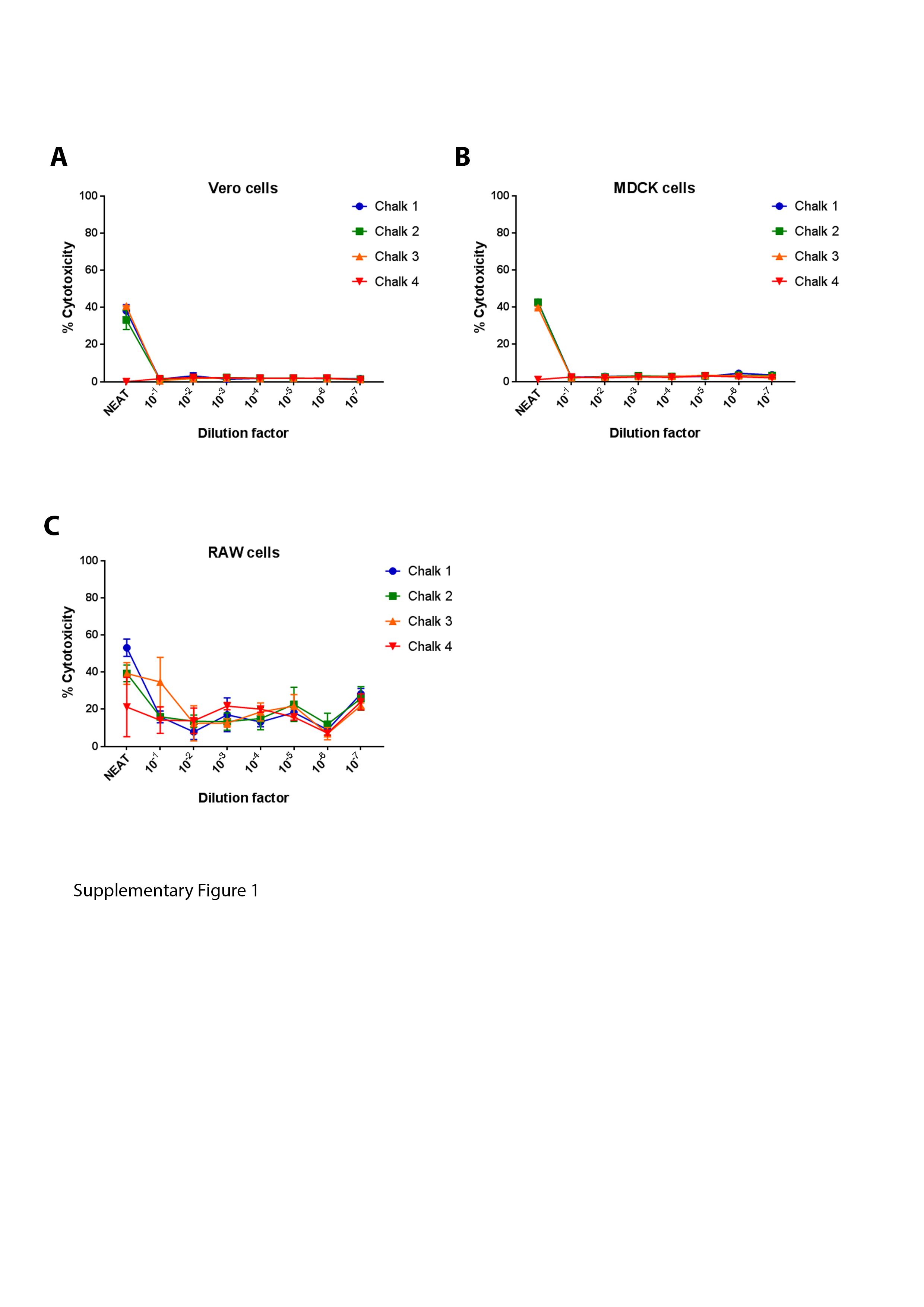

Supplement: FIG S1 [file msphere.00313-21-sf001.tif]

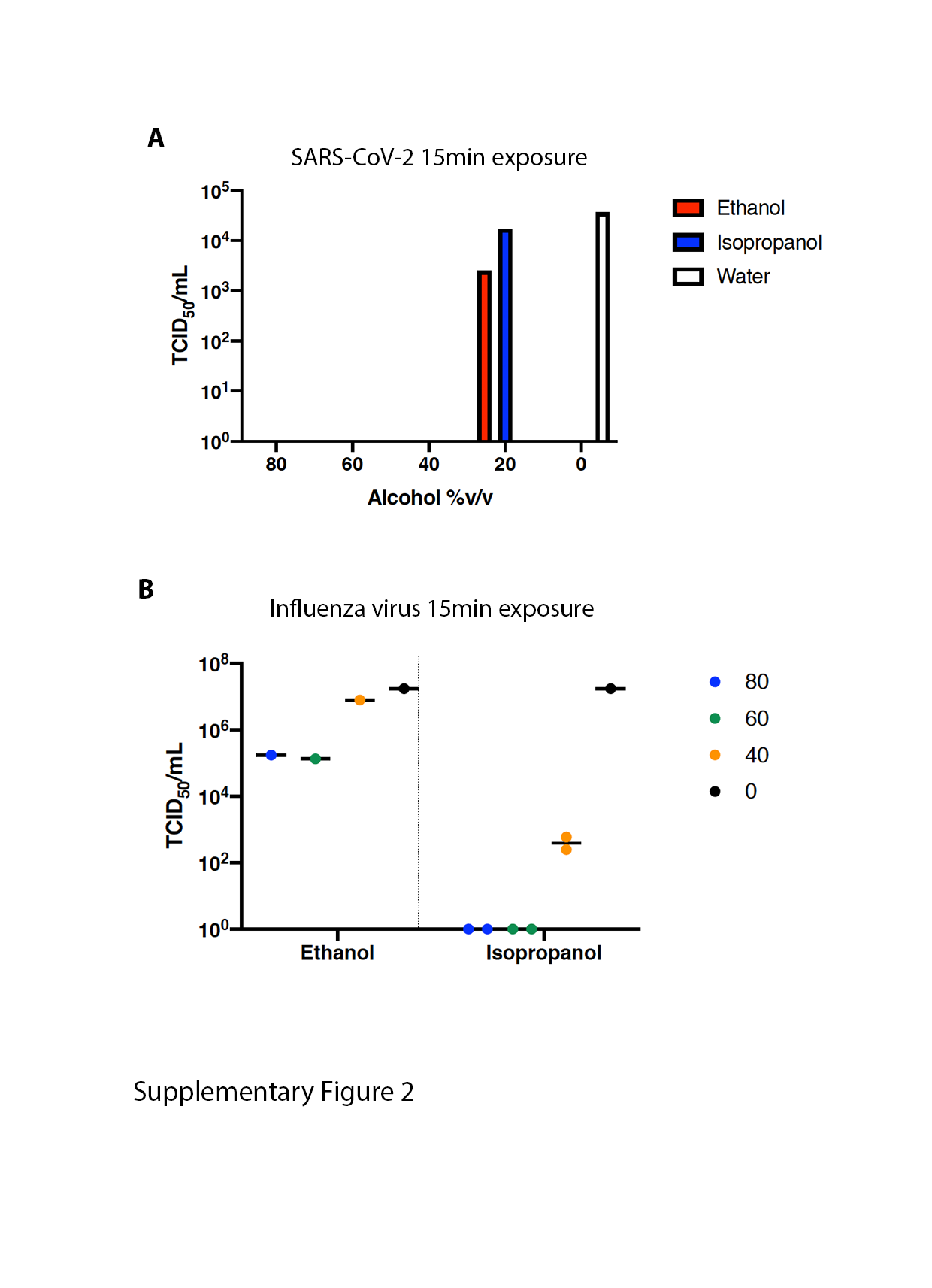

Supplement: FIG S2 [file msphere.00313-21-sf002.tif]
