# Supplementary material for: Liquid Chalk Is an Antiseptic against SARS-CoV-2 and Influenza A Respiratory Viruses
Source: mSphere. 2021 Jun 16;6(3):e00313-21. doi: 10.1128/mSphere.00313-21 (PMC8265647; doi:10.1128/mSphere.00313-21)
Supplement: TEXT S1 [file msphere.00313-21-t0001.docx]

**Supplementary Material**

**Liquid chalk is an antiseptic against pathogenic enveloped human respiratory viruses**

Julie L. McAuley^1^, Joshua M. Deerain,^1^ Turgut E. Aktepe^1^, William Hammersla^2^, Damian J.F. Purcell^1^ and Jason M. Mackenzie^1*^

^1^Department of Microbiology and Immunology, University of Melbourne, at the Peter Doherty Institute for Infection and Immunity, Parkville, VIC 3010; and ^2^Urban Climb, Collingwood, VIC 3066, Australia

^*^Corresponding author: phone +613 9035 8376

email [jason.mackenzie@unimelb.edu.au](mailto:jason.mackenzie@unimelb.edu.au)

Keywords: SARS-CoV-2, COVID-19, influenza A virus, Norovirus, antiseptic, alcohol

**Materials and Methods:**

*Cell culture maintenance and virus stocks:*

Vero cells (American Type Culture Collection [ATCC]) were maintained in Minimal Essential Media (MEM) supplemented with 10% heat-inactivated foetal bovine serum (FBS), 10 μM HEPES, 2 mM glutamine and antibiotics ((100 units/mL Penicillin G, 100 µg/mL Streptomycin). Madin-Darby canine kidney (MDCK) cells were grown in Roswell Park Memorial Institute (RPMI) media supplemented with 10% FBS, 2 mM glutamine and antibiotics. RAW 264.7 cells were maintained in Dulbecco’s Modified Eagle’s Medium (DMEM) with 10% FBS and 1% GlutaMAX. Cell cultures were maintained at 37^o^C in a 5% CO_2_ incubator. SARS-CoV-2 isolate hCoV-19/Australia/VIC01/2020 (*1*) stocks, were produced as previously described (*2*). The influenza A virus isolate, A/Puerto Rico/8/34 was produced as previously described (*3*). Details of MNV strain CW1 have been described previously (*4, 5*).

*Cytotoxicity assay 96® Non-Radioactive Cytotoxicity Assay (Promega):*

Vero, MDCK and RAW cells were plated in a 96-well plate to 80% confluency. 50ul of various liquid chalk samples were aseptically air-dried, resuspended in 500ul of cell culture media and centrifuged at 400xg for 3 mins to remove excess chalk particles (this sample is depicted as “neat”). Neat supernatant was 10-fold serially diluted in respective tissue culture media, added to the 96-well plate containing cells and incubated at 37ᴼC for 24 hours. Following the incubation period, 10ul of 10x Lysis Solution was added for 45 mins to the control wells to generate a Maximum LDH Release Control. 50ul of supernatant from each sample (in duplicates) was transferred to a fresh 96-well flat clear bottom plate and incubated for 30 mins with 50ul of CytoTox 96® Reagent. To end the reaction, 50ul of Stop Solution was added to each well and absorbance was recorded at 490nm.

Percent cytotoxicity = 100 x (Experimental LDH Release (OD490)/Maximum LDH Release (OD490))

*Chalk exposure assays:*

All SARS-CoV-2 infection cultures were conducted within the High Containment Facilities in a PC3 laboratory at the Doherty Institute. For the chalk first assay, sample of the liquid chalk was aseptically smeared onto 2-4 discrete areas covering approximately 2cm round surface (approx. 50ul) on a sterile tissue culture dish and allowed to dry. 50uL virus inoculum was then applied. Where the inoculum did not absorb into the dry chalk, a slurry of chalk:virus mixture was created using a sterile tip and mixing. 15min later, 500uL infection media (identical to culture media but without the presence of sera) was added then mixed with the chalk:virus sample and collected. Excess chalk was pelleted at 400 g for 3min, then a 50% tissue culture infectious dose (TCID50) assay performed on the supernatant as previously described (*2, 3*). For the virus first assay, 50uL virus inoculum was added to 2-4 discrete areas on a sterile tissue culture dish, then chalk added and spread to cover an approximate 2cm round surface on a sterile tissue culture dish. After 15min incubation at room temperature, 500uL infection media was added and sample mixed, then remaining virus present in the sample quantitated in the same way as per the chalk first procedure. For both tests, no chalk control received 50uL infection media.

*Statistical Analyses:*

Data is representative of at least 2 independent experiments and was analysed using GraphPad Prism v8.0.

**Supplementary Results:**

*Cytotoxicity assays:*

The Vero, MDCK and RAW264.7 cells used in this study will all exposed to differing dilutions of the liquid chalks to determine the cytotoxic effect of on the different cell types. As can be observed in Supplementary Figure 1, only when the liquid chalks were used at a neat (or 100%) dilution did the cells show any adverse or cytotoxic effects. Even after just a 1:10 dilution the cytotoxic effects were dramatically reduced int eh Vero and MDCK cells. This was more evident in the 1”100 dilution for RAW264.7 cells. All of these dilutions are within the limits used during the treatment studies and indicate that the chalk itself is not contributing to the effects observed.

In addition, as we had observed inhibitory effects with the liquid chalks against both SARS-CoV-2 and IAV we next determined the impact of alcohol treatment alone on each of these viruses. As can be observed in Supplementary Figure 2, 80% and 60% ethanol affected the infectivity of IAV but 40% did not. Comparatively isopropanol even at 40% caused a highly significant loss of infectivity.

**References:**

1. L. Caly *et al.*, Isolation and rapid sharing of the 2019 novel coronavirus (SARS-CoV-2) from the first patient diagnosed with COVID-19 in Australia. *Med J Aust* **212**, 459-462 (2020).

2. J. Y. H. Lee *et al.*, Validation of a single-step, single-tube reverse transcription loop-mediated isothermal amplification assay for rapid detection of SARS-CoV-2 RNA. *J Med Microbiol*, (2020).

3. J. L. McAuley *et al.*, Expression of the 1918 influenza A virus PB1-F2 enhances the pathogenesis of viral and secondary bacterial pneumonia. *Cell Host Microbe* **2**, 240-249 (2007).

4. S. M. Karst, C. E. Wobus, M. Lay, J. Davidson, H. W. Virgin, IV, STAT1-Dependent Innate Immunity to a Norwalk-Like Virus. *Science* **299**, 1575-1578 (2003).

5. J. L. Hyde *et al.*, Mouse norovirus replication is associated with virus-induced vesicle clusters originating from membranes derived from the secretory pathway. *J Virol* **83**, 9709-9719 (2009).
